# Unveiling of a puzzling dual ionic migration in lead‐ and iodide‐deficient halide perovskites (d‐HPs) and its impact on solar cell *J–V* curve hysteresis

**DOI:** 10.1002/EXP.20220156

**Published:** 2023-10-20

**Authors:** Liam Gollino, Daming Zheng, Nicolas Mercier, Thierry Pauporté

**Affiliations:** ^1^ Chimie‐ParisTech, PSL Université, CNRS Institut de Recherche de Chimie‐Paris (IRCP) Paris cedex 05 France; ^2^ MOLTECH‐Anjou University of Angers Angers France

**Keywords:** glow discharge optical emission spectroscopy, halide perovskite, hysteresis, iodide‐deficiency, ionic migration, lead‐deficiency, solar cells

## Abstract

Halide perovskite solar cells are characterized by a hysteresis between current–voltage (*J*–*V)* curves recorded on the reverse and on the forward scan directions, and the suppression of this phenomenon has focused great attention. In the present work, it is shown that a special family of 3D perovskites, that are rendered lead ‐and iodide‐ deficient (d‐HPs) by incorporating large organic cations, are characterized by a large hysteresis. The strategy of passivating defects by K^+^, which has been successful in reducing the hysteresis of 3D perovskite perovskite solar cells, is inefficient with the d‐HPs. By glow discharge optical emission spectroscopy (GD‐OES), the existence of the classic iodide migration in these layers is unveiled, which is efficiently blocked by potassium cation insertion. However, it is also shown that it co‐exists with the migration of the large organic cations characteristics of d‐HPs which are not blocked by the alkali metal ion. The migration of those large cations is intrinsically linked to the special structure of the d‐HP. It is suggested that it takes place through channels, present throughout the whole perovskite layer after the substitution of PbI^+^ units by the large cations, making this phenomenon intrinsic to the original structure of d‐HPs.

## Graphical abstract

In the present paper, a phenomenon of ionic migration of large organic cations in the lead‐ and iodide‐deficient perovskite films is unveiled. This phenomenon is intrinsic to the structure and at the origin of a large hysteresis in the measured *J*–*V* curves. Contrary to iodide, it cannot be fully blocked through defect passivation by potassium ions.

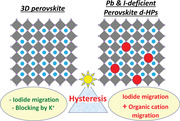

## 1 INTRODUCTION

The operation under light of halide perovskite solar cells (PSCs) is characterized by a hysteresis between current–voltage (*J*–*V)* curves recorded in the reverse scan direction and in the forward scan direction. Classically, the reverse scan is located above the forward one. It results in a higher power conversion efficiency (PCE) determined on the former compared to the latter. PSC *J*–*V* hysteresis was first reported in 2014 by Unger et al.^[^ ^1^ ^]^ Many studies have been dedicated to this puzzling effect since then,^[^ ^2^, ^3^, ^4^ ^]^ and four main hypotheses on its origin have been put forward: the ferroelectric effect,^[^ ^4^, ^5^, ^6^, ^7^, ^8^, ^9^ ^]^ the unbalanced charge transport/extraction between holes and electrons,^[^ ^10^, ^11^, ^12^ ^]^ the trap‐assisted charge recombination,^[^ ^13^, ^14^ ^]^ and the ionic migration.^[^ ^15^, ^16^, ^17^ ^]^ (i) The ferroelectric effect is the spontaneous electric polarization induced under the application of an electric field. In hybrid halide perovskite (PVK), it is linked to the presence of polar molecules, such as MA^+^ in MAPbI_3_. Because of this effect, after the reverse scan (to measure PSCs efficiency), an excess polarization would exist in the device and lead to a decreased *J*_SC_ during the forward scan. However, the ferroelectric effect is under debate and is questioned as the source of hysteresis in PSCs. While some groups reported a relationship between ferroelectric polarization and hysteresis,^[^ ^18^, ^19^, ^20^ ^]^ various researchers found that the ferroelectric domain relaxation is too quick (<1 ms) compared to *J*–*V* curve measurement (up to several minutes),^[^ ^21^, ^22^ ^]^ being thus not linked to the hysteresis phenomenon.^[^ ^23^, ^24^, ^25^ ^]^ (ii) The unbalanced charge transport/extraction is caused by the unequal diffusion lengths and mobilities of charge carriers in the PSCs. If these two parameters are not correctly paired, it will cause a charge carrier accumulation either near electron transport layer (ETL)/PVK or hole transport layer (HTL)/PVK interfaces. Ultimately, it will result in a capacitance phenomenon that possibly produces hysteresis. (iii) The trap‐assisted charge recombination has its origin in the defects within the PSCs (perovskite bulk, grain boundaries, ETL/PVK or PVK/HTL interfaces) leading to trap states. During the forward scan measurement, charge carriers are trapped within the defects/trap states and are gradually released when the scan is reversed. Such a release of charge carriers may induce a photocurrent and voltage delay and thus hysteresis between reverse and forward measurements. (iv) In PSCs, ions can migrate under polarization and accumulate near the charge transport layers, causing hysteresis under operation conditions. The main explanation remains the ionic migration occurring within the perovskite layer. Many groups reported the importance of ionic migration by observing slow transient effects.^[^ ^26^, ^27^, ^28^ ^]^ The quasi‐reversible field assisted halide migration was revealed on a lateral cell using a synchrotron‐based nanoprobe X‐ray fluorescence technique.^[^ ^29^ ^]^ It also showed a vacancy‐mediated migration using DFT simulation combined with photoluminescence (PL).^[^ ^29^ ^]^ However, direct experimental evidence was provided later. Glow‐discharge optical emission spectroscopy (GD‐OES) technique allowed the follow‐up of iodide migration through the perovskite film thickness under the application of an electric field.^[^ ^30^, ^31^ ^]^ In MAPbI_3−_ *_x_* Cl*_x_* perovskite, Lee et al. observed iodide and chloride migrations through polarization while lead (Pb^2+^) and organic cations (MA^+^, FA^+^) remained static.^[^ ^32^, ^33^ ^]^ This observation was consistent with theoretical calculations of activation energy for ion migration of each species ^[^ ^24^, ^34^, ^35^, ^36^, ^37^ ^]^. Recently, Pauporté et al. explained more precisely the link between iodide migration within the perovskite and hysteresis by correlating iodide migration and the value of the hysteresis amplitude.^[^ ^30^ ^]^

Beyond the well‐studied 3D and 2D hybrid halide perovskites, an additional hybrid perovskite family, named d‐HPs, has been reported.^[^ ^38^, ^39^, ^40^, ^41^, ^42^, ^43^ ^]^ Lead and iodide‐deficient MAPbI_3_ and FAPbI_3_ have been prepared using hydroxyethylammonium HO─(CH_2_)_2_─NH_3_^+^ (HEA^+^)^[^ ^38^, ^39^, ^40^, ^41^ ^]^ and thioethylammonium HS─(CH_2_)_2_─NH_3_^+^ (TEA^+^)^[^ ^39^, ^40^, ^41^ ^]^ organic cations and have been integrated in PSCs. d‐HP thin films based on MAPbI_3_ and iodoethylammonium I─(CH_2_)_2_─NH_3_^+^ (IEA^+^), on one side,^[^ ^42^ ^]^ and on the dication 2‐hydroxypropane‐1,3‐diaminium ^+^NH_3_CH_2_CH(OH)CH_2_NH_3_^+^, (PDA^2+^) and MAPbI_3_ and FAPbI_3_, on the other side,^[^ ^43^ ^]^ have been reported by our groups recently. These materials retain a 3D structure while inserting rather large cations in their lattice. In ref. [38, 39, 40, 41, 42, 43], both experimental and theoretical structural studies of these compounds have concluded to an original 3D structure in which PbI^+^ units are replaced by the large cation, leading to the general formula (A, A’)_1+_ *_x_* Pb_1−_ *_x_* I_3−_ *_x_* (A = MA^+^ or FA^+^; A’ = HEA^+^, TEA^+^, IEA^+^ or PDA^2+^). They have been prepared as crystals, powders, and crystallized thin films by spin‐coating.^[^ ^38^, ^39^, ^40^, ^41^, ^42^, ^43^ ^]^ All d‐HP based PSCs exhibited *J*–*V* curves with a large hysteresis.

Similar structures have also been reported by Kanatzidis et al. based on the use of other large cations such as ethylenediammonium,^[^ ^44^, ^45^, ^46^, ^47^, ^48^, ^49^, ^50^ ^]^ propylenediammonium, and trimethylenediammonium,^[^ ^51^ ^]^ and this family has been called “Hollow” perovskites. These new 3D materials are promising for optoelectronic applications, not only because of their reduced lead content, but also in view of the large flexibility of their chemical composition.

This article focuses on the MAPbI_3_‐HEA and the FAPbI_3_‐TEA systems. We first present an investigation of the *J*–*V* curves of solar cells prepared with d‐HP absorber layers. We show that they are characterized by a large hysteresis which follows the same scan rate dependence as the classical 3D perovskite solar cells (PSCs). Glow discharge optical emission spectroscopy (GD‐OES) technique combined with time‐resolved photoluminescence (TRPL) has been then implemented to follow the species which are mobile under an electric field. Large organic cation used to form d‐HPs has been found to migrate within the layer, in an opposite direction to I^−^, creating a coexistence of two different migrating species.

## 2 RESULTS AND DISCUSSION

We tested a large number of parameters for each system and the present work implements the optimized ones. We selected an *x* = 0.13 value (MA_0.68_HEA_0.45_)Pb_0.87_I_2.87_,^[^ ^38^, ^39^ ^]^ named d‐MAPI‐HEA) for the d‐MAPI‐HEA system and a *x* = 0.04 value (TEA_0.12_FA_0.92_)Pb_0.96_I_2.96_,^[^ ^39^ ^]^ named d‐FAPI‐TEA)for the d‐FAPI‐TEA one. Such x values were shown, in our previous reports^[^ ^38^, ^39^, ^40^, ^41^ ^]^ and preliminary studies, to give the best compromise between lead and iodide reduction, efficiency, and stability while retaining the d‐HP structure.

**FIGURE 1:**
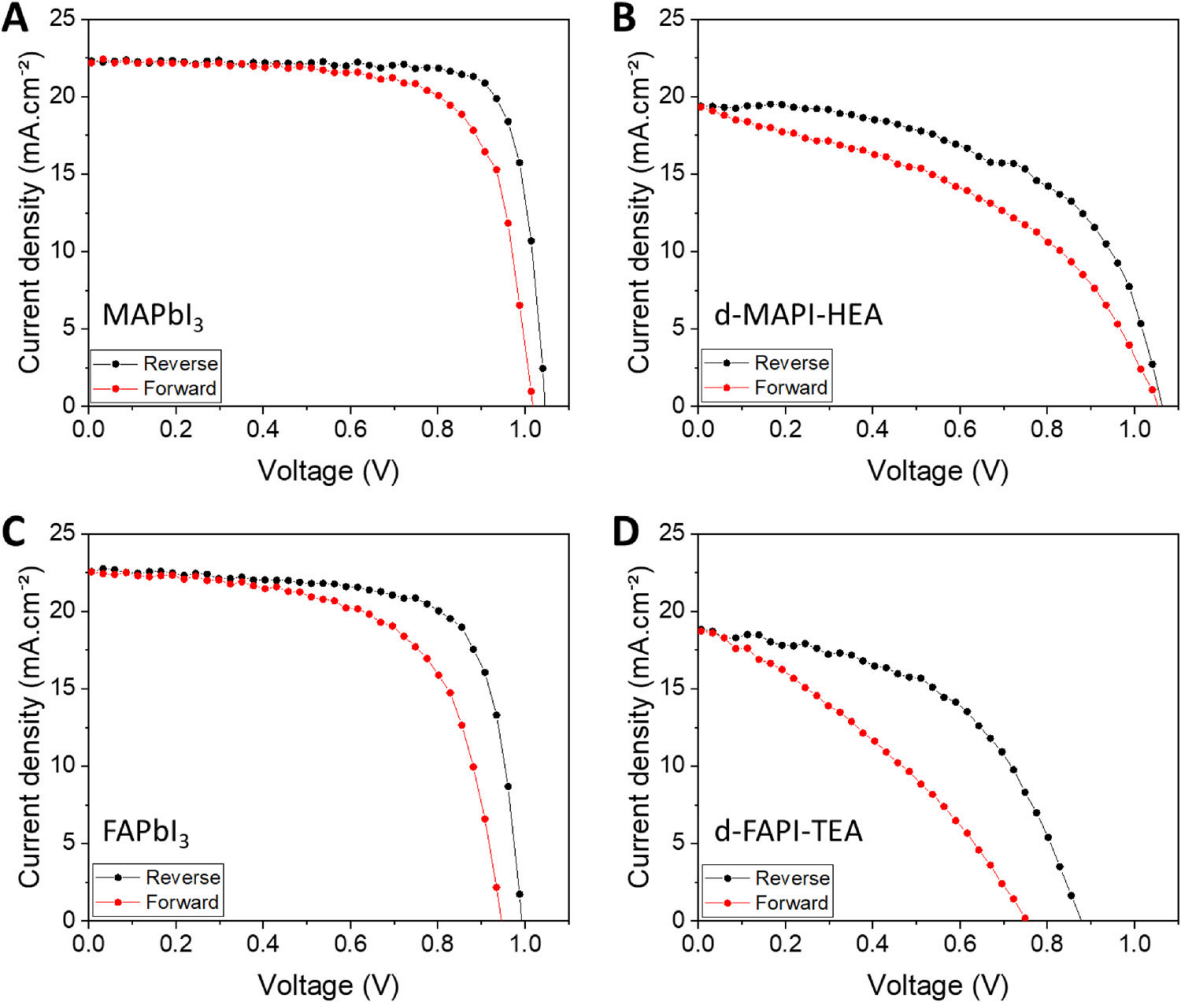
A, MAPbI_3_, B, d‐MAPI‐HEA, C, FAPbI_3_, and D, d‐FAPI‐TEA devices (measured with a scan rate of 0.14 V s^−1^).

Figure 1 compares typical *J–V* curves of FTO/c‐TiO_2_/m‐TiO_2_/perovskite/Spiro‐OMeTAD/Au PSCs using classical 3D MAPI and FAPI PVK absorbers and two d‐HPs, one containing MA^+^ and HEA^+^ cations and the other prepared with FA^+^ and TEA^+^ cations. The associated *J–V* parameters and PCE are gathered in Table S1. In spite of many optimizations implemented in our group,^[^ ^40^ ^]^ the efficiency of the two d‐HPs remains far below the ones of 3D‐HPs with 11.5 % and 8.3 % for d‐MAPI‐HEA and d‐FAPI‐TEA, respectively Table S1, Supporting Information). Another feature of the d‐HP PSCs is that they exhibit a larger hysteresis. Hysteresis has been quantified by the hysteresis index (HI) defined as:

(1) $$\mathrm{HI}\left(\%\right)=\frac{\left({\mathrm{PCE}}_{\mathrm{rev}}-{\mathrm{PCE}}_{\mathrm{for}}\right)\times 100}{{\mathrm{PCE}}_{\mathrm{rev}}}$$

with PCE_rev_ and PCE_for_ being the PCE measured on the reverse scan and forward scan, respectively. While classic 3D MAPbI_3_ and FAPbI_3_‐based PSCs exhibit rather low HI values of 14.5 % and 17.9 %, respectively, the d‐MAPI‐HEA and d‐FAPI‐TEA ones have larger values of 39.4% and 43.4%, respectively Table S1. The insertion of large organic cations within MAPbI_3_ and FAPbI_3_ lattices during the formation of the d‐HPs seems thus to be detrimental for the hysteresis. We then investigated the effect of the scan rate (*v*) on the HI parameter. Typical curves are gathered in Figure S1. A similar shape is found for 3D HPs and d‐HPs. They are characterized by a minimum at 0.02–0.04 V s^−1^, HI quickly increases then to a maximum at 0.05–0.14 V s^−1^ before to slowly decrease with *v*. It is related to ion migration which induces an electric field that causes itself interfacial ion accumulation and has opposite direction depending on forward and reverse scans ^[^ ^52^, ^53^, ^54^, ^55^ ^]^. For a slow scan rate, ions have sufficient time to accumulate at the interface. Therefore, the built‐in electric field is completely screened by the ion‐induced electric field, making the *J–V* characteristics that are close for reverse and forward scans and then the hysteresis is weak. For a fast scan rate, ions do not have enough time to migrate so there is less ions accumulation. It results in a non‐apparent built‐in electric field for reverse and forward scans and then a weak hysteresis once again. For an in‐between scan rate (close to the system response scan rate), it exists a large difference in ion density between reverse and forward scans, i.e. a difference in electric field which causes a larger *HI* value than for slower or faster scan rates. In view of the high HI recorded for d‐HPs solar cells, we tested the employment of KCl as a soluble additive compound in the perovskite precursor solution. K^+^ has been shown to passivate the defects in the perovskite layer through which I can migrate.^[^ ^31^ ^]^ It results in small hysteresis in prepared 3D perovskite‐based PSCs. This beneficial effect has been shown for Cs_0.1_FA_0.9_PbI_3_ and FAMAPbI_3_ perovskites for instance in ref. [30] results are given in Tables S2 and S3 for various KCl concentrations employed for the preparation of d‐MAPI‐HEA. Unfortunately, KCl did not improve the performance of the device and the hysteresis remained high. In the case of d‐FAPI‐TEA, we found a drop in the PCE and an unchanged HI Table S4 and Figure S2. To unveil the puzzling behaviour of d‐HP, further investigations were implemented by glow discharge optical emission spectroscopy (GD‐OES).

GD‐OES is a powerful analytical technique that provides elemental depth profiles of layered structures. It is of special interest for the fast analysis of thin film‐based devices such as perovskite solar cells.^[^ ^31^, ^56^, ^57^ ^]^ Figures S3 shows the GD‐OES profile of the two reference PVK solar cells. Figure S4 and S5 show GD‐OES profiles of 3D d‐HP solar cells. Au of the back contact is observed at the surface (pale yellow background) and the perovskite layer is localized from Pb and I traces (pale red background). The Ti signal appears later, in the perovskite layer domain because the mesoporous TiO_2_ ETL layer is filled with perovskite. In the case of d‐FAPI‐TEA PSC, sulphur was detected in the perovskite film (Figure S5). This element, which is the signature of TEA^+^, was homogeneously distributed throughout the perovskite film. On the other hand, HEA^+^ cation (HO‐(CH_2_)_2_‐NH_3_^+^) could not be distinguished by our GD‐OES system, since nitrogen and carbon elements, are also present in MA^+^ of the perovskite.

**FIGURE 2:**
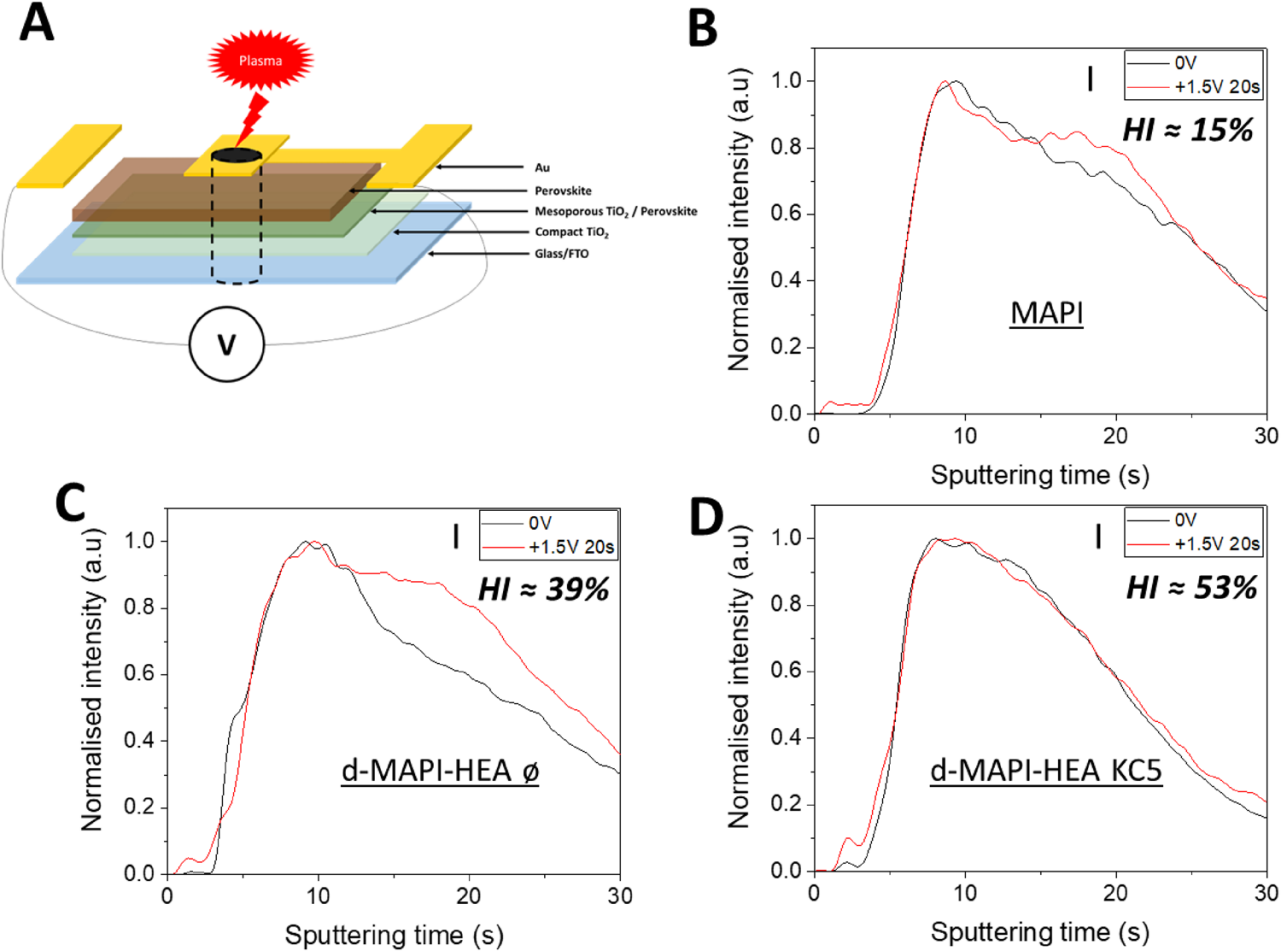
GD‐OES working principle and curve profiles. A, Schematic illustration of the architecture of devices used to perform GD‐OES measurements. The plasma beam penetrating progressively into the layers is depicted by black dashed lines. The 1.5 V battery used during polarization and ionic migration study is also shown. B–D, Normalized GD‐OES iodide profile curves with polarization at 0 and +1,5 V for 20 s. B, MAPbI_3_, C, d‐MAPI‐HEA, and D, d‐MAPI‐HEA PSCs prepared with 5% KCl. The curves are normalized to highlight the shift of I profiles. The HI value of the PSCs are indicated in the figures in B–D.

The next step was to polarize the cells just before to measure the GD‐OES profile in order to observe the changes induced by the electric field (Figure 2A). We first followed the I^−^ distribution across the perovskite layers after a polarization at 0 and at +1.5 V for 20 s. The corresponding GD‐OES profiles are shown in Figures 2B and 2C for MAPbI_3_ and d‐MAPI‐HEA, respectively. In both cases, we observed a splitting of the I profile with clearly a part of I which migrates toward the FTO electrode. For the MAPbI_3_, that possesses a rather small hysteresis of 15 %, just a small shift in the iodide profile toward the right, i.e. the FTO electrode, was observed. It is consistent with the negative charge of the I^−^ ion (Figure 2B). The shift in the iodide profile was larger for d‐MAPI‐HEA without KCl that possesses a higher HI value of about 39 % (Figure 2C). The correlation between the increased iodide migration and the higher HI value is in line with our previous observations on other systems.^[^ ^30^ ^]^ The GD‐OES profiles prove the existence of iodide migration in d‐MAPI‐HEA perovskite.

We then investigated d‐MAPI‐HEA KC5, prepared with 5% KCl, by GD‐OES (Figure S4B). The K profile presented a homogeneous distribution throughout the entire perovskite layer thickness (Figure S4C). We have also noted a Cl signal of the same amplitude as the noise (Figure S4C). GD‐OES is an extremely sensitive technique, therefore a signal in the noise signifies an amount of concerned element less than 0.1 at.%, which, in our case, indicates the complete elimination of Cl^−^ upon annealing due to low sublimation temperature of Cl^−^ compounds. After addition of KCl in d‐MAPI‐HEA, we saw that the iodide profiles measured after a polarization at 0 V and at 1.5 V overlapped (Figure 2D). This overlapping means that the iodide migration was successfully blocked by the passivation of defects by K^+^. The effect of KCl on d‐MAPI‐HEA hysteresis values are summarized in Tables S2 and S3, for the best cells and average, respectively. They show that this blocking does not suppress the *J–V* curves hysteresis and that *HI* remained high in the case of d‐HPs.

**FIGURE 3:**
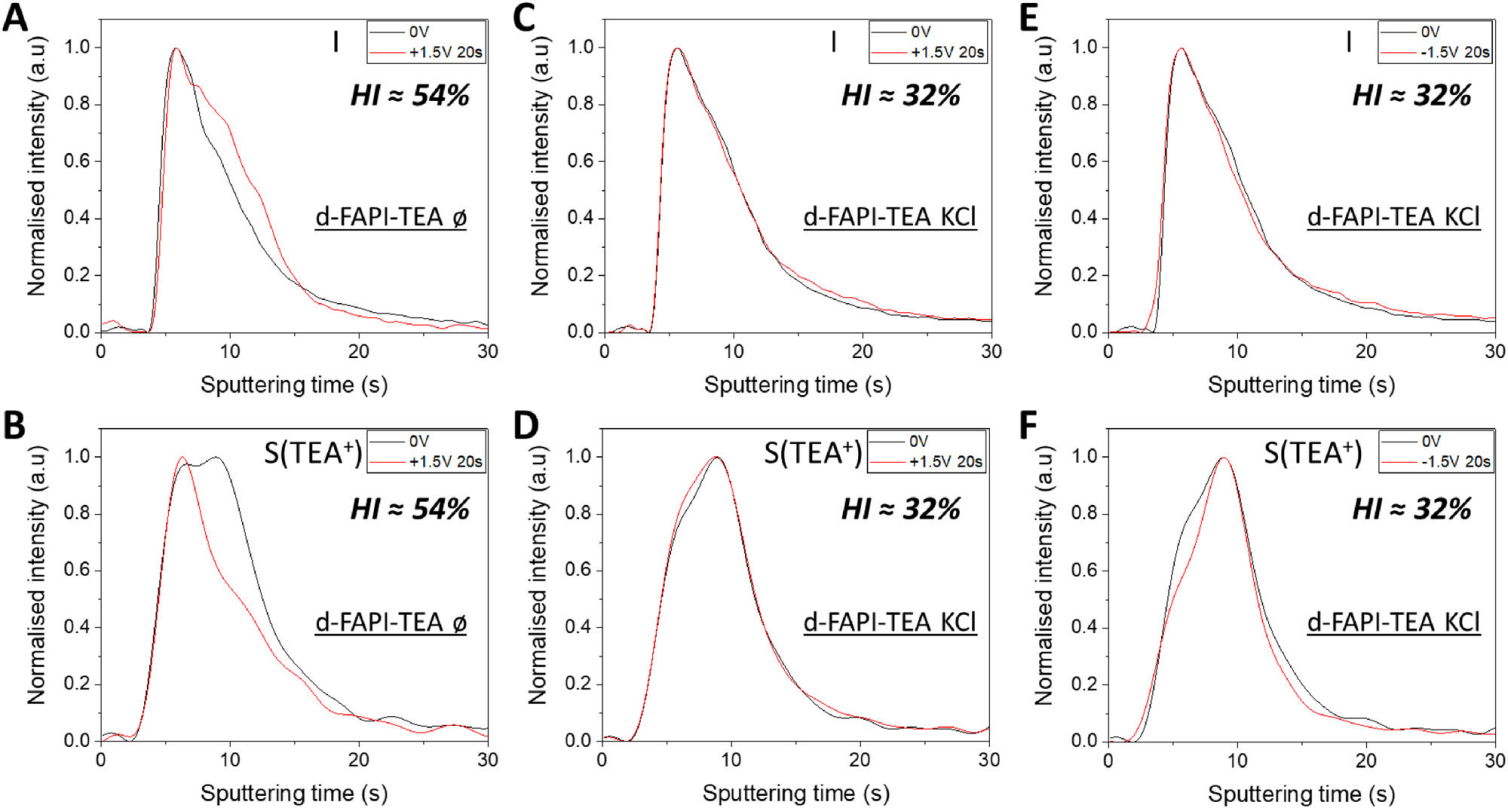
Polarization effects on the GD‐OES profiles. A,C,E, Profiles of iodide and B,D,F, TEA^+^ of d‐FAPI‐TEA before (black trace) and after (red trace) polarization. A, B, Pristine cell profiles with a polarization of 0 V and +1.5 V. C–F, Profiles for cells prepared with 5 mol% of KCl with a polarization of 0and +1.5 V C,D, and with a polarization of 0 V and −1.5 V E,F. The HI value of the PSCs are indicated in the figures.

We carried out a similar study for the d‐FAPI‐TEA samples (Figure 3 and Figure S5). SEM and XRD characterizations of the investigated films are provided in Figure S6. After application of a positive bias, the I trace was right‐shifted for the pristine sample. On the other hand, the two traces overlapped for the sample with KCl additive. In this case, we also investigated a negative applied bias (Figure 3E,F). The two traces were also overlapping, demonstrating the efficient iodide blocking. However, HI remained high since it was only reduced from 54% to 32 %. The puzzling result with the d‐MAPI‐HEA and d‐FAPI‐TEA systems is that if I^−^ migration is blocked by K^+^ in both cases, their HI*s* remain high. This suggests that the additional organic cation species present in the d‐HPs may be free to migrate in the structure.

In the investigated devices, we removed the Spiro‐OMeTAD layer, which contains sulphur in Li‐TFSI additive. Consequently, TEA^+^ cation was the only compound with sulphur left in the device. The S signal corresponds to the TEA^+^ signal. By looking at the TEA^+^ profiles (Figure 3B), an important shift to the left is observed after the positive polarization, meaning a migration of the TEA^+^ cations towards the Au electrode. The migration direction, opposite to the I^−^ one, is in adequacy with the positive charge of the cation. It proves the co‐existence of two migrating species within d‐FAPI‐TEA perovskite: I^−^ and TEA^+^. To add further proof and comprehend more deeply the importance of TEA^+^ migration within d‐FAPI‐TEA perovskite, the same experiment was performed with KCl treated cells. We investigated a positive (Figure 3D) and a negative polarization (Figure 3E). By applying a positive polarization to the cell, a small shift of the TEA^+^ profile to the left was observed, indicating a way smaller migration than the one observed for pristine cell. Therefore, the TEA^+^ migration is reduced by K^+^ but not suppressed. The migration towards the Au electrode is difficult to observe as for low sputtering time, the GD‐OES plasma is still unstable, leading to some shape effects for the signals at such low sputtering time. We also investigated the application of a negative polarization (Figure 3F). A larger shift was then observed compared to the one measured with a positive polarization. With this additional experiment, the proof of existence of TEA^+^ migration (and its co‐existence with I^−^ migration) within d‐FAPI‐TEA perovskite was shown. We have seen that the TEA^+^ profile is homogeneously spread throughout the perovskite thickness (Figure S5). This proves that the PbI^+^ units substitution by TEA^+^ cations would occur identically over the entirety of the perovskite layer and not at specific place. We can then suppose that the created network is continuous and allows the displacement of TEA^+^ which is not blocked by K^+^ because, in its presence, the d‐HP structure is preserved. The TEA^+^ mobility and electric field feeling would be the reason behind the important HI value in d‐FAPI‐TEA.

**FIGURE 4:**
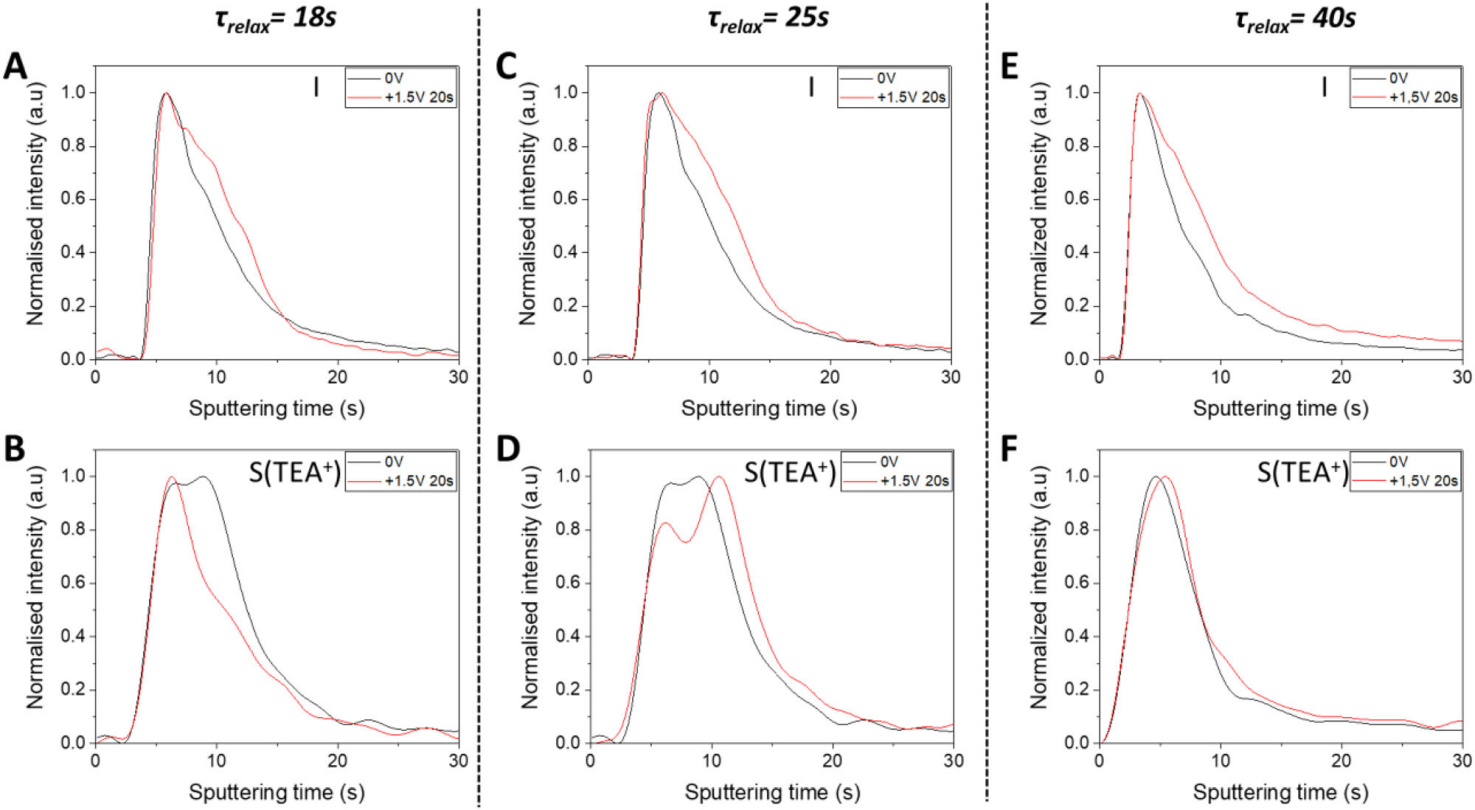
Relaxation effects on the GD‐OES profiles. GD‐OES profile of iodide A,C,E, and TEA^+^ B,D,F, for a relaxation time of 18 s A,B, 25 s C,D, and 40 s (E,F) before (black trace) and after (red traces) polarization at +1.5 V.

We then investigated the migration speed of I^−^ and TEA^+^. We repeated the previous experiment on K^+^‐free cells, but now the delay between the 20 s long polarization and the GD‐OES measurement of the cells was varied. This delay is named relaxation time and is noted *τ*_relax_ hereafter. We followed the evolution of the profile for I^−^ and TEA^+^ ions. The relaxation time was measured precisely from the moment the pristine cell (without KCl) was disconnected from the 1.5 V battery and the moment when the GD‐OES profile measurement started. We set three different *τ*_relax_ at 18, 25, and 40 s, to be able to determine the relative migration speeds of the two mobile ions within the perovskite and link them to their role in the hysteresis of the final devices. The results are displayed in Figure 4. Regarding TEA^+^, for a short *τ*_relax_ of 18 s, it migrates towards the Au electrode in agreement with its positive charge. For a higher *τ*_relax_ of 25 s, the profile is split into two peaks indicating two parts of TEA^+^: one still localized in the upper perovskite layer part and another fraction that has moved to recover its initial position. The splitting of the TEA^+^ profile indicates a clear relaxation of these ions between 18 and 25 s after polarization and the rather rapid movement of TEA^+^. For a *τ*_relax_ of 40 s, the black and red curves overlap meaning that the polarization effect on TEA^+^ has been cancelled. This cation has fully relaxed and has almost returned to its initial position then. On the other hand, iodide profiles did not significantly change upon the first 40 s of *τ*_relax_. All these results indicate a rather fast relaxation of TEA^+^ ions, while the I^−^ ions have a slow relaxation after polarization stopping.

Our study concludes of a correlation between the HI value and the ionic migration. However, it seems that the part of hysteresis caused by TEA^+^ is as or more important than the part of I^−^. This result is very surprising in view of the small amount of TEA^+^ presents within the perovskite lattice (11% of organic cations). However, it could be explained by the presence of migration channels within the perovskite lattice created by TEA^+^ insertion. In fact, for a various amount of substitution (x < 0.20), two hypotheses can be made, either a partial filling of all channels by PbI^+^ units of the reference structure (*x* = 0.20 – channels only filled by organic cations‐), or the full filling of some channels by PbI^+^ units, some channels remaining lead and iodide‐free (Figure S7). We favoured this last hypothesis in ref. [39], and the results obtained here fit with this hypothesis and would explain the ease for cations to migrate through the whole perovskite layer. We indeed proved that the TEA^+^ is homogeneously present in the entirety of the perovskite layer thickness (Figure S5). With the presence of such channels in the structure and throughout the whole layer, it becomes rather easy for cations such as TEA^+^ to migrate over the whole layer thickness and then finally cause important hysteresis in operating devices. The significantly higher I^−^ anion amount compared to TEA^+^ cation (d‐FAPI‐TEA = (TEA_0.12_FA_0.92_)Pb_0.96_I_2.96_) has to be considered and cannot be neglected. This will undeniably create an important built‐in potential in the PSCs that will attract the TEA^+^ in the opposite direction of their initial migration once the electric field is shut off. It will cause them to rapidly come back to their initial position or a bit moved towards the FTO electrode. Indeed, the right shift observed after relaxing 25 s (Figure 4D) (accentuated by the normalization of the profile) would be then a consequence of the slower relaxation of iodide and to the negative charge accumulated near the ETL side. Such a phenomenon can also explain the fast relaxation measured for TEA^+^.

**FIGURE 5:**
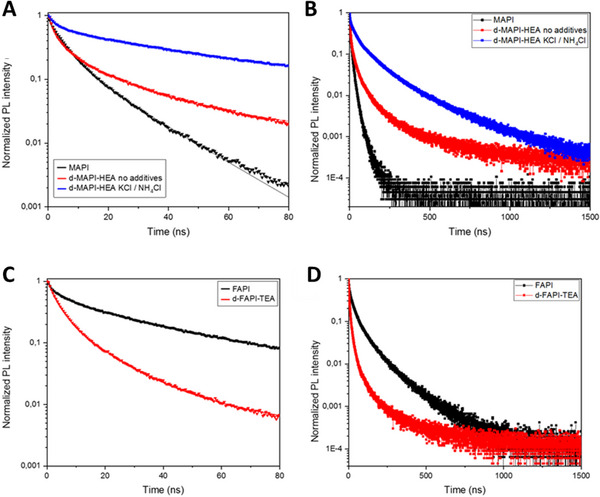
Normalized time‐resolved photoluminescence of PVK and d‐HP samples. A, MAPbI_3_ (black trace) and d‐MAPI‐HEA with and without additives (blue and red traces) films on FTO/c‐TiO_2_ substrates measured up to 80 ns. The full lines are the fit curves. B, Same as A, measured up to 1500 ns. C, Normalized time‐resolved photoluminescence (TRPL) of FAPbI_3_ (black trace) and d‐FAPI‐TEA (red trace) films on FTO/c‐TiO_2_ substrates measured up to 80 ns. D, Same as C, measured up to 1500 ns. The full lines are the fit curves.

TRPL measurements were also conducted using FTO/c‐TiO_2_/m‐TiO_2_/PVK substrates. The obtained decay curves were fitted using a triple exponential function to get the best coefficient of determination *R* ^2^. The decay curves are depicted in Figure 5 while the used triple exponential function and the obtained extracted fitting parameters are gathered in Table S5. Using such a fitting especially allows the extraction of the parameter named *τ*_fast_, that represents the non‐radiative recombination at the ETL/PVK interface which reflects the speed of charge injection in the ETL. The lower the *τ*_fast_ value, the better the charge extraction, therefore, lower charge accumulation and interfacial recombination are expected. d‐MAPI‐HEA exhibits a higher *τ*_fast_ value of 2.60 ns compared to MAPbI_3_ with a *τ*_fast_ value of 0.72 ns (≈ 4 times higher). A similar trend is observed for d‐FAPI‐TEA which disclosed 5.3 times higher *τ*_fast_ than FAPbI_3_ (12.29 ns versus 2.31 ns). Such an increase of *τ*_fast_ reveals a downgrading of the ETL/PVK interface for both d‐MAPI‐HEA and d‐FAPI‐TEA, compared to their classic 3D MAPbI_3_ and FAPbI_3_ analogues. Such a lower quality of this interface is responsible for a poor electron extraction by TiO_2_ that leads to a charge accumulation that will exacerbate the hysteresis of the devices (ii) phenomenon in the introduction).

## 3 CONCLUSION

In conclusion, our comprehensive investigation of d‐HP based PSCs has allowed us to understand the origin of the large HI measured on these devices that strongly hinders their potential and development. The large cations (HEA^+^, TEA^+^) inserted in 3D perovskites to form d‐HPs appear to migrate effectively within the perovskite layer, as well as I^−^ ions, causing the co‐existence of two ionic species migrations in the PSCs in opposite directions. This dual migration complexify greatly the hysteresis cancelation compared to classic 3D perovskites. It renders the use of d‐HP in solar cells problematic due to low performances. We have shown the efficient I^−^ blocking by K^+^ and we have hypothesized that the organic cation migration occurs through what we call migration channels that exist due to the special structure of d‐HPs in which PbI^+^ units are substituted by large organic cations. The substitution was found to occur throughout the entirety of the perovskite layer thickness, allowing the cation to move across these channels in the whole PVK layer. Moreover, we have shown that they have a faster relaxation than the I^−^ anions which follow other pathways. It is attributed to the substantially higher number of I^−^ species compared to the big organic cations that will attract them towards the other side of the cell once the external electric field is shut off. The hysteresis problem is then intrinsic to the d‐HPs and to date, it seems extremely complex to get rid of it. In addition, TRPL measurements suggest a poor electron charge extraction by TiO_2_ that will lead to charge accumulation near the interface and exacerbate the hysteresis of the devices.

## 4 EXPERIMENTAL METHODS

### 4.1 Materials

Methylammonium iodide (MAI, 99.99%), formamidinium iodide (FAI, 99.99%), anatase TiO_2_ NR30‐D paste, and tris(2‐1H‐pyrazol‐1‐yl)−4‐tert‐butylpyridine)‐cobalt(III) tri(bis(trifluoromethylsulfonyl)imide) (FK209 salt) were purchased from Greatcell Solar Materials. Lead iodide (PbI_2_, 99.99%) was obtained from TCI Europe N.V. Dimethyl sulfoxide (DMSO, 99.9% anhydrous), *N*,*N*‐dimethyl formamide (DMF, 99.9% anhydrous), 1‐methyl‐2‐pyrrolidone (NMP, 99.5% anhydrous), chlorobenzene (99.8% anhydrous), potassium chloride (KCl, 99.5%), ammonium chloride (NH_4_Cl, 99.5%), 4‐tert‐butylpyridine (tBP, 99.0%) and bis(trifluoromethane) sulfonimide lithium salt (Li‐TFSI, 99.0%) were bought from Sigma Aldrich. Titanium(IV) isopropoxide 99.995% was purchased from Thermoscientific. SpiroOMeTAD (99.8%) was bought from Borun New Material Technology Ltd. All these chemicals were used as received without further purification. 2.2 mm thick glass‐FTO substrate (Pilkington TEC 8) was purchased from Xop Glass. Hydroxyethylammonium iodide (HEAI) was prepared as described in ref. [38]. Thioethylammonium iodide was prepared as described in ref. [39].

### 4.2 Layers and devices preparation

The substrates were cut, etched, and cleaned as described elsewhere.^[^ ^56^ ^]^ A dense and thin TiO_2_ blocking layer was then deposited by spray pyrolysis and a TiO_2_ mesoporous layer was deposited by spin‐coating.^[^ ^57^, ^58^ ^]^ 3D and d‐HP perovskite films were subsequently deposited.

#### 4.2.1 MAPbI_3_ perovskite

A perovskite precursor solution with a concentration of 1.35 m was prepared in a N_2_ filled glovebox by mixing MAI (214.6 mg) and PbI_2_ (622.3 mg) in DMSO (1 mL). The bottle was tightly capped, and the solution was stirred for 2 h at 100°C. In a N_2_‐filled glovebox, we placed 40 μL of this solution on top of the substrate before starting the spin‐coating routine. The program used was: 1000 rpm with an acceleration of 200 rpm s^−1^ for 10 s followed by a second spinning at 6000 rpm with an acceleration of 4000 rpm s^−1^ for 30 s. 100 μL of chlorobenzene was dripped 30 s after the start of the spinning routine at a slow speed using a manual micropipette. The films were then annealed on a hotplate at 105°C for 1 h under a N_2_ atmosphere. The final mesoporous TiO_2_/ MAPbI_3_ perovskite layer average thickness was about 510 nm.

#### 4.2.2 d‐MAPI‐HEA perovskites

A perovskite precursor solution with a concentration of 1 m was prepared in a N_2_ filled glovebox by dissolving HEAI (59.7 mg), MAI (143 mg), PbI_2_ (461 mg) in a mixture of DMF (900 μL) and DMSO (100 μL). For the samples with 5 mol% of KCl, we added KCl (3.7 mg) and NH_4_Cl (15 mg) to the solution. The solution was stirred for 4 h at 50°C in a N_2_‐filled glovebox. 40 μL of this solution was deposited on top of the substrate before starting the spin‐coating routine. A two‐step spin‐coating program was used: first spinning at 1000 rpm with an acceleration of 500 rpm s^−1^ for 10 s followed by a second spinning at 5000 rpm with an acceleration of 1500 rpm s^−1^ for 30 s. For the quenching, 100 μL of chlorobenzene was dripped 15 s after the start of the spinning routine using an electronic micropipette Eppendorf Xplorer with a defined and optimized ejection speed. The films were finally annealed at 105°C for 1 h under a N_2_ atmosphere.

For the study of KCl effect on the hysteresis, the same protocol was employed, except that various amounts of KCl were added to the precursor solution and NH_4_Cl was removed. For precursor solutions with 5 / 9 / 13 mol% KCl, 3.7 / 6.7 / 9.7 mg of KCl were added. The final mesoporous TiO_2_/ d‐MAPI‐HEA perovskite layer average thickness was about 545 nm.

#### 4.2.3 FAPbI_3_ perovskite

A perovskite precursor solution with a concentration of 1.2 m was prepared in a N_2_‐filled glovebox by mixing FAI (206 mg) and PbI_2_ (553 mg) in a solvent mixture of DMF (800 μL) and DMSO (200 μL). The solution was stirred for 2 h at room temperature. About 40 μL of this solution was placed on top of the substrate before starting the spin‐coating. A two‐step spin‐coating program was used: first spinning at 1000 rpm with an acceleration of 1000 rpm s^−1^ for 10 s to homogeneously spread the solution. Then the spinning was risen to 6000 rpm with an acceleration rate of 4000 rpm s^−1^ for 30 s. 100 μL of chlorobenzene was dripped 20 s after the start of the spinning routine using an electronic micropipette Eppendorf Xplorer with a defined and optimized ejection speed. The films were then annealed at 153°C for 13 min under a N_2_ atmosphere.^[^ ^59^ ^]^ The final mesoporous TiO_2_/ FAPbI_3_ perovskite layer average thickness was about 570 nm.

#### 4.2.4 d‐FAPI‐TEA perovskites

A perovskite precursor solution with a concentration of 1.1 м was prepared in a N_2_‐filled glovebox by mixing TEAI (37.6 mg), FAI (189.1 mg) and PbI_2_ (507 mg) in a solvent mixture of DMF (800 μL) and NMP (200 μL). The solution was stirred for 4 h at 50°C. About 40 μL of this solution was deposited on top of the substrate before starting the spin‐coating. A two‐step spin‐coating program was used: first spinning at 1000 rpm for 10 s with an acceleration of 200 rpm s^−1^ followed by a second spinning at 6000 rpm for 20 s with an acceleration of 4000 rpm s^−1^. 100 μL of chlorobenzene was dripped 15 s after the start of the spinning routine using an electronic micropipette Eppendorf Xplorer with a defined and optimized ejection speed. The films were then annealed at 125°C for 30 min under a N_2_ atmosphere.

For the study of KCl on the hysteresis, the same protocol was used except that of KCl (4.1 mg, 5 mol%) was added to the precursor solution and no NH_4_Cl was added. The final mesoporous TiO_2_/ d‐FAPI‐TEA perovskite layer average thickness was about 540 nm.

For the *J–V* curve measurement devices, a Spiro‐OMeTAD (HTM) layer was deposited by spin‐coating as described before.^[^ ^30^ ^]^ A gold back electrode was evaporated under vacuum.^[^ ^59^ ^]^ The Spiro‐OMeTAD layer was not present for the GD‐OES migration studies.

### 4.3 Devices characterizations

Glow Discharge Optical Emission Spectroscopy (GD‐OES) analyses were performed using a HORIBA Jobin Yvon GD Profiler 2 equipment. This instrument was equipped with a RF‐generator (at 13.56 MHz), a standard HORIBA Jobin Yvon glow discharge source with a cylindrical anode of 4 mm internal diameter and two optical spectrometers (a polychromator and a monochromator) for fast‐optical detection. The argon (Ar) plasma was generated at an Ar gas pressure of 420 Pa and an applied power of 17 W. The solar cell was mounted on an O‐ring at one side of the plasma chamber and used as a cathode. For the polarization experiment, a 1.5 V battery was connected to the samples with the help of crocodile clamps right next to the GD‐OES apparatus. The cell was short circuited for the 0 V experiment. After polarization, the sample was placed in the GD‐OES chamber and the measurement was started. The time between the polarization end and the GD‐OES measurement start was about 15 s due to the chamber pumping and a pre‐integration time inherent to the apparatus. The TRPL curves were measured as described before.^[^ ^60^ ^]^

The solar cells *J–V* curves were recorded by a Keithley 2410 digital source meter, using a voltage scan rate ranging between 0.02 V s^−1^ and 0.4 V s^−1^. The solar cells were illuminated with a solar simulator (Abet Technology Sun 2000) filtered to mimic AM 1.5G conditions (100 mW cm^−2^).^[^ ^61^ ^]^ The illuminated surface was delimited by a black mask with an aperture diameter of 3 mm. The power density was calibrated at 100 mW cm^−2^ by the use of a reference silicon solar cell.

More experimental details are given in the Supporting Information.

## AUTHOR CONTRIBUTIONS

The manuscript was written through contributions of Liam Gollino and Thierry Pauporté. Liam Gollino, Daming Zheng, and Thierry Pauporté carried out the laboratory experimental research on perovskite films and solar cells including all the associated characterizations. Nicolas Mercier provided the HEAI and TEAI precursors. All authors read and amended the final version of the manuscript. All authors have approved the final version of the manuscript.

## CONFLICT OF INTEREST STATEMENT

The authors declare no conflicts of interest.

## Supporting information

Complementary experimental information on PSCs fabrication. *J–V* parameters, PCE and *HI* of best MAPbI_3_, d‐MAPI‐HEA, FAPbI_3_ and d‐FAPI‐TEA devices. Evolution of MAPbI_3_, FAPbI_3_ and d‐MAPI‐HEA‐based PSCs hysteresis as a function of the scan rate used for the *J–V* measurements. *J–V* parameters, PCE and HI of best d‐MAPI‐HEA devices with different KCl amount (MAPbI_3_ shown as reference). Average photovoltaic *J–V* parameters, PCE and HI of d‐MAPI‐HEA devices with different KCl amounts (MAPbI_3_ cells are shown as references). Average photovoltaic *J–V* curve parameters and PCE with standard deviation of d‐FAPI‐TEA devices with and without KCl (7 cells per system). Box charts of *V*_OC_, *J*_SC_, FF, PCE, and HI parameters of d‐FAPI‐TEA PSCs without and with 5 mol% KCl (noted KC5). 7 cells per system. GD‐OES profiles of MAPbI_3_, FAPbI_3_ devices. d‐MAPI‐HEA devices (with and without KCl additives). GD‐OES profile of Cl and K in d‐MAPI‐HEA KC5 perovskite film. GD‐OES profiles of d‐FAPI‐TEA KC5 device with Cl and K profiles. SEM top‐view image of d‐FAPI‐TEA (pristine and with KCl) films with associated XRD patterns. Structure of d‐FAPI‐TEA. Fit results of time‐resolved photoluminescence curves of MAPbI_3_, d‐MAPI‐HEA, FAPbI_3_, and d‐FAPI‐TEA films on FTO/c‐TiO_2_/m‐TiO_2_ substrates.

## Data Availability

The data are available on request from the authors.
