# Supplementary material for: Unveiling of a puzzling dual ionic migration in lead‐ and iodide‐deficient halide perovskites (d‐HPs) and its impact on solar cell J–V curve hysteresis
Source: Exploration (Beijing). 2023 Oct 20;4(1):20220156. doi: 10.1002/EXP.20220156 (PMC10867389; doi:10.1002/EXP.20220156)
Supplement: Supplementary file 1 — Complementary experimental information on PSCs fabrication. J–V parameters, PCE and HI of best MAPbI3, d‐MAPI‐HEA, FAPbI3 and d‐FAPI‐TEA devices. Evolution of MAPbI3, FAPbI3 and d‐MAPI‐HEA‐based PSCs hysteresis as a function of the scan rate used for the J–V measurements. J–V parameters, PCE and HI of best d‐MAPI‐HEA devices with different KCl amount (MAPbI3 shown as reference). Average photovoltaic J–V parameters, PCE and HI of d‐MAPI‐HEA devices with different KCl amounts (MAPbI3 cells are shown as references). Average photovoltaic J–V curve parameters and PCE with standard deviation of d‐FAPI‐TEA devices with and without KCl (7 cells per system). Box charts of V OC, J SC, FF, PCE, and HI parameters of d‐FAPI‐TEA PSCs without and with 5 mol% KCl (noted KC5). 7 cells per system. GD‐OES profiles of MAPbI3, FAPbI3 devices. d‐MAPI‐HEA devices (with and without KCl additives). GD‐OES profile of Cl and K in d‐MAPI‐HEA KC5 perovskite film. GD‐OES profiles of d‐FAPI‐TEA KC5 device with Cl and K profiles. SEM top‐view image of d‐FAPI‐TEA (pristine and with KCl) films with associated XRD patterns. Structure of d‐FAPI‐TEA. Fit results of time‐resolved photoluminescence curves of MAPbI3, d‐MAPI‐HEA, FAPbI3, and d‐FAPI‐TEA films on FTO/c‐TiO2/m‐TiO2 substrates. [file EXP2-4-20220156-s001.docx]

Supporting Information

Unveiling of a Puzzling Dual Ionic Migration in Lead- and Iodide-Deficient Halide Perovskites (d-HPs) and its Impact on Solar Cell *J-V* Curve Hysteresis

Liam Gollino^1^, Daming Zheng^1^, Nicolas Mercier^2^ and Thierry Pauporté*^1^

*^1^Chimie-ParisTech, PSL Université, CNRS, Institut de Recherche de Chimie-Paris (IRCP), UMR8247, 11 rue Pierre et Marie Curie, F-75231 Paris cedex 05, France*

*^2^University of Angers, MOLTECH-Anjou, UMR 6200, 2 boulevard de Lavoisier, 49045 Angers*

*Corresponding author

*T.Pauporté (*[*thierry.pauporte@chimieparistech.fr*](mailto:thierry.pauporte@chimieparistech.fr)*)*

*Chimie-ParisTech, PSL Université, CRNS, Institut de Recherche de Chimie-Paris (IRCP), UMR8247, 11 rue Pierre et Marie Curie, F-75231*

**Complementary Experimental**

Fluorine-doped SnO_2_ (FTO) substrates were etched pattern by zinc oxide powder and 10% HCl solution prior to be cleaned with soap and water. The substrates were subsequently plunged for 20 min in a concentrated 2.2 м NaOH in ethanol/water (10:1 volume ratio), rinsed with water, cleaned in acetone using an ultrasonic bath for 12 min and then rinsed with deionized water in an ultrasonic bath for 15 min. The substrates were then heated at 500 °C for 15 min. The compact TiO_2_ electron transporting layer (ETL), noted c-TiO_2_, was prepared by aerosol spray pyrolysis. The mesoporous TiO_2_ ETL, noted m-TiO_2_ was prepared using a nanoparticle solution made in advance and stirred at least 12 h. The anatase TiO_2_ NR30-D paste was diluted in ethanol with a 1:7 wt/wt ratio. 45 μL of the solution was dropped on the compact TiO_2_ layer and spin-coated at 2000 rpm for 15 s. The layer was then dried on a hotplate at 70 °C for at least 10 min and finally heated at 500 °C under an air flux for 30 min, cooled down to 200 °C and removed from the hotplate before being transferred immediately to a N_2_-filled glovebox for perovskite layer deposition.

The hole transporting layer (HTL) solution was prepared by dissolving spiro-OMeTAD (78 mg) in chlorobenzene (1 mL). Then, Li-TFSI solution (17.9 μL) (prepared by dissolving Li-TFSI (517 mg) in acetonitrile (1 mL)), of tBP (30.4 μL), and tris(2-1H-pyrazol-1-yl)-4-tert-butylpyridine)-cobalt(III) tri(bis(trifluoromethylsulfonyl)imide) (14 μL) (376 mg of FK209 in 1 mL acetonitrile) were added to this solution. About 40 μL of the HTM solution was spin coated at 4000 rpm for 30 s. Finally, a back-electrode was deposited by thermally evaporating a 70–80 nm-thick gold layer on the spiro-OMeTAD layer. For GD-OES devices, the gold back electrode was deposited directly on the perovskite layer.

**Table S1** *J-V* parameters*, PCE* and *HI* of best MAPbI_3_, d-MAPI-HEA, FAPbI_3_ and d-FAPI-TEA devices.

| Sample | Scan direction | V_OC_ (V) | J_SC_ (mA.cm^-^²) | FF (%) | PCE (%) | HI (%) |
| --- | --- | --- | --- | --- | --- | --- |
| MAPI | Reverse | 1.04 | 22.29 | 81.44 | 18.94 | **14.5** |
|  | Forward | 1.02 | 22.33 | 71.29 | 16.20 |  |
| d-MAPI-HEA | Reverse | 1.06 | 19.36 | 55.76 | 11.47 | **39.4** |
|  | Forward | 1.05 | 19.54 | 42.96 | 8.82 |  |
| FAPbI_3_ | Reverse | 0.99 | 22.63 | 72.40 | 16.23 | **17.9** |
|  | Forward | 0.95 | 22.52 | 62.56 | 13.33 |  |
| d-FAPI-TEA | Reverse | 0.88 | 18.68 | 50.79 | 8.33 | **43.4** |
|  | Forward | 0.75 | 18.89 | 33.13 | 4.71 |  |


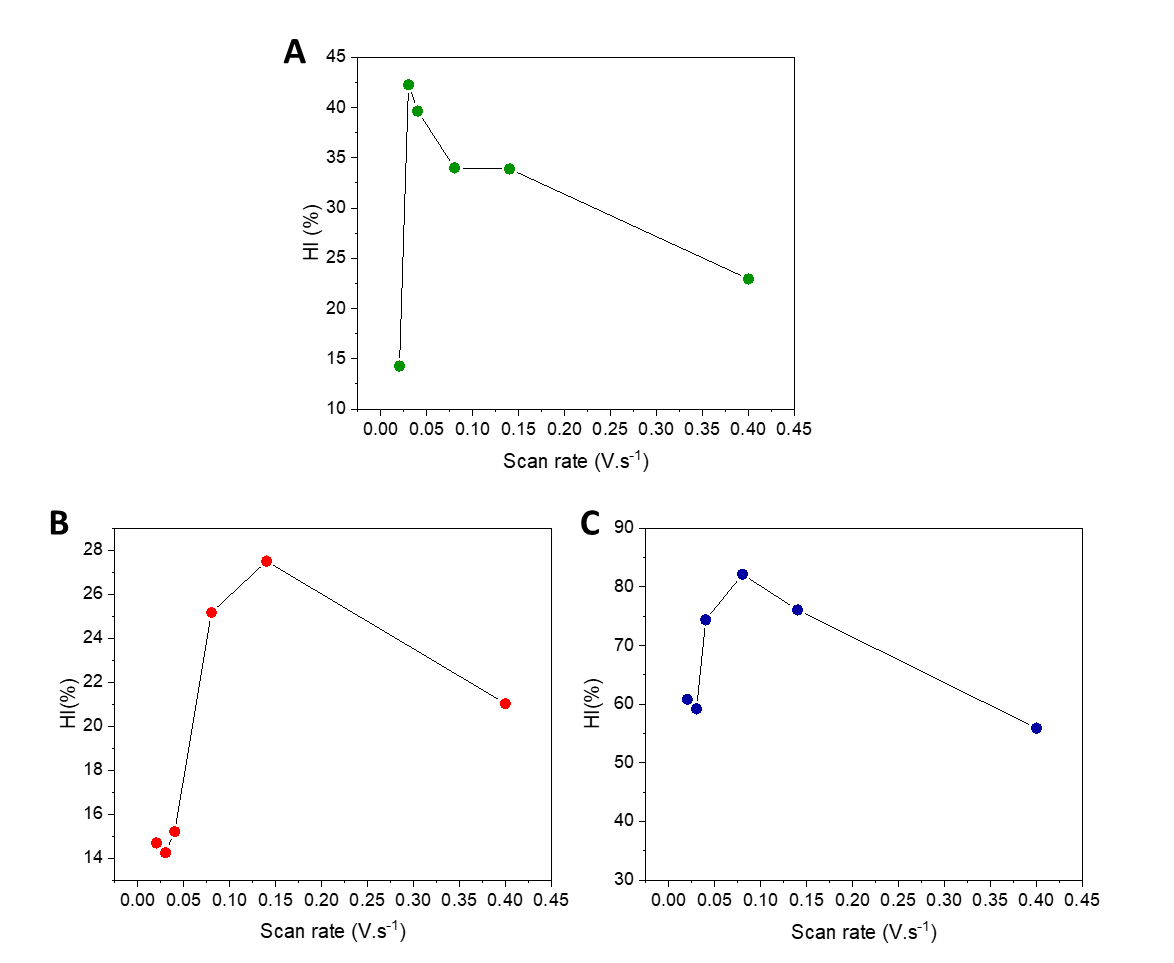


**Figure S1.** Hysteresis index as a function of the scan rate used for the *J-V* measurements. (A) FAPbI_3_, (B) MAPbI_3_, and (C) d-MAPI-HEA (optimized with KCl/NH_4_Cl co-additives)

**Table S2** *J-V* parameters*, PCE* and *HI* of best MAPI and d-MAPI-HEA devices with different KCl amounts.

| Sample | Scan direction | V_OC_ (V) | J_SC_ (mA.cm^-^²) | FF (%) | PCE (%) | HI (%) |
| --- | --- | --- | --- | --- | --- | --- |
| MAPbI_3_ | Reverse | 1.04 | 22.29 | 81.44 | 18.94 | **14.5** |
|  | Forward | 1.02 | 22.33 | 71.29 | 16.20 |  |
| d-MAPI-HEA KC0 | Reverse | 1.05 | 13.51 | 66.56 | 9.48 | **39.4** |
|  | Forward | 1.02 | 13.52 | 41.51 | 5.74 |  |
| d-MAPI-HEA KC5 | Reverse | 0.98 | 14.78 | 65.71 | 9.53 | **54.1** |
|  | Forward | 0.85 | 13.61 | 37.69 | 4.38 |  |
| d-MAPI-HEA KC9 | Reverse | 0.92 | 15.83 | 61.16 | 8.93 | **48.8** |
|  | Forward | 0.83 | 14.23 | 38.89 | 4.57 |  |
| d-MAPI-HEA KC13 | Reverse | 0.87 | 16.29 | 60.73 | 8.60 | **55.7** |
|  | Forward | 0.78 | 15.12 | 32.34 | 3.81 |  |

**Table S3** Average photovoltaic *J-V* parameters*, PCE* and *HI* of MAPI and d-MAPI-HEA devices with different KCl amounts (4 cells per system).

| Sample | Scan direction | V_OC_ (V) | J_SC_ (mA.cm^-^²) | FF (%) | PCE (%) | HI (%) |
| --- | --- | --- | --- | --- | --- | --- |
| MAPbI_3_ | Reverse | 1.05 ± 0.01 | 22.29 ± 0.49 | 78.74 ± 1.93 | 18.46 ± 0.33 | **15.1 ± 3.3** |
|  | Forward | 1.00 ± 0.02 | 22.28 ± 0.41 | 70.02 ± 2.30 | 15.68 ± 0.78 |  |
| d-MAPI-HEA KC0 | Reverse | 1.01 ± 0.06 | 12.80 ± 1.00 | 59.89 ± 9.89 | 7.83 ± 2.33 | **47.2 ± 11.0** |
|  | Forward | 0.93 ± 0.13 | 12.10 ± 2.01 | 36.43 ± 7.19 | 4.26 ± 2.09 |  |
| d-MAPI-HEA KC5 | Reverse | 0.95 ± 0.04 | 14.86 ± 0.42 | 61.98 ± 4.95 | 8.73 ± 0.92 | **54.3 ± 1.4** |
|  | Forward | 0.82 ± 0.03 | 13.33 ± 0.58 | 36.30 ± 2.61 | 3.99 ± 0.41 |  |
| d-MAPI-HEA KC9 | Reverse | 0.92 ± 0.01 | 14.90 ± 1.15 | 60.20 ± 1.83 | 8.24 ± 0.58 | **48.2 ± 5.7** |
|  | Forward | 0.83 ± 0.02 | 12.92 ± 1.29 | 33.67 ± 2.79 | 4.27 ± 0.55 |  |
| d-MAPI-HEA KC13 | Reverse | 0.87 ± 0.02 | 15.26 ± 1.24 | 61.11 ± 2.35 | 8.10 ± 0.60 | **56.3 ± 5.3** |
|  | Forward | 0.77 ± 0.04 | 13.43 ± 1.93 | 34.52 ± 5.38 | 3.55 ± 0.56 |  |

**Table S4.** Average photovoltaic *J-V* curve parameters, *PCE* and *HI* with standard deviation of d-FAPI-TEA devices with and without KCl (7 cells per system).

| Sample | Scan direction | V_OC_ (V) | J_SC_ (mA.cm^-^²) | FF (%) | PCE (%) | HI (%) ^(a)^ |
| --- | --- | --- | --- | --- | --- | --- |
| d-FAPI-TEA pristine | Reverse | 0.84 ± 0.05 | 15.91 ± 1.21 | 49.41 ± 1.34 | 6.61 ± 0.86 | 54.2 ± 7.7 |
|  | Forward | 0.73 ± 0.05 | 15.47 ± 1.51 | 26.71 ± 3.96 | 3.04 ± 0.77 |  |
| d-FAPI-TEA KC5 | Reverse | 0.82 ± 0.03 | 3.59 ± 0.38 | 60.85 ± 1.38 | 1.78 ± 0.18 | 41.7 ± 9.5 |
|  | Forward | 0.76 ± 0.05 | 3.15 ± 0.50 | 43.85 ± 5.19 | 1.05 ± 0.24 |  |


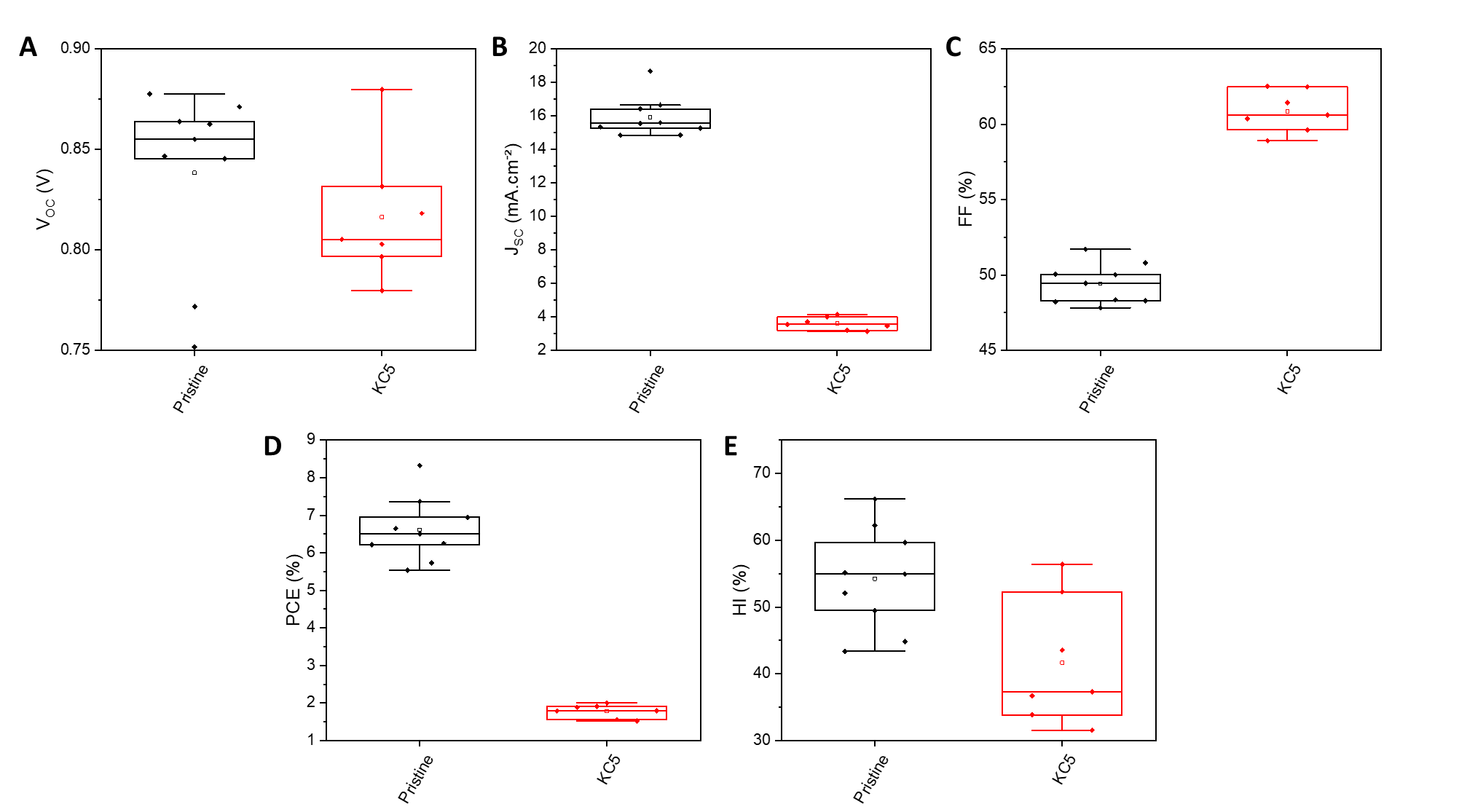


**Figure S2.**  Box charts of (A) *V_OC_*, (B) *J_SC_*, (C) *FF*, (D) *PCE,* and (E) *HI* parameters of d-FAPI-TEA PSCs without and with 5 mol% KCl (noted KC5). 7 cells per system.

**
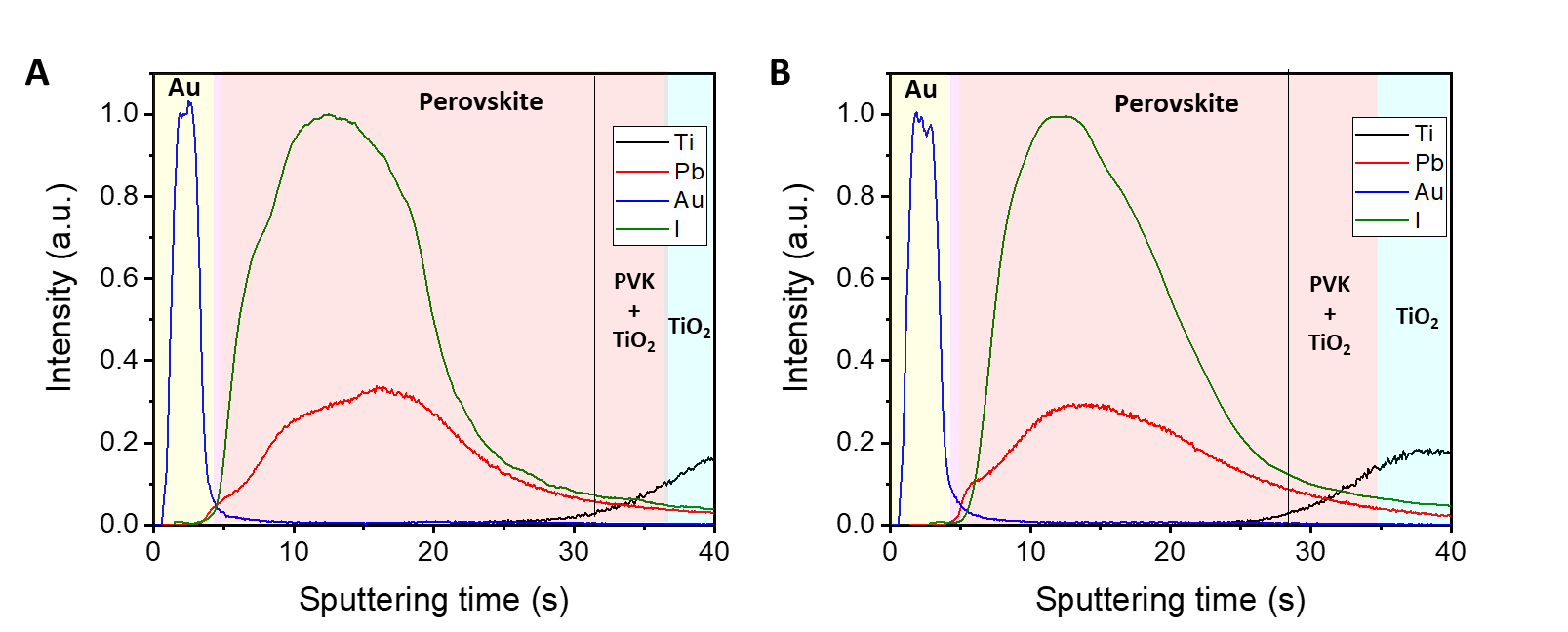
**

**Figure S3.** GD-OES profile of complete (A) MAPbI_3_ and (B) FAPbI_3_ solar cells. The colored backgrounds identify the layers: yellow : gold back contact; purple : SpiroOMeTAD; Salmon : perovskite; blue : TiO_2_.


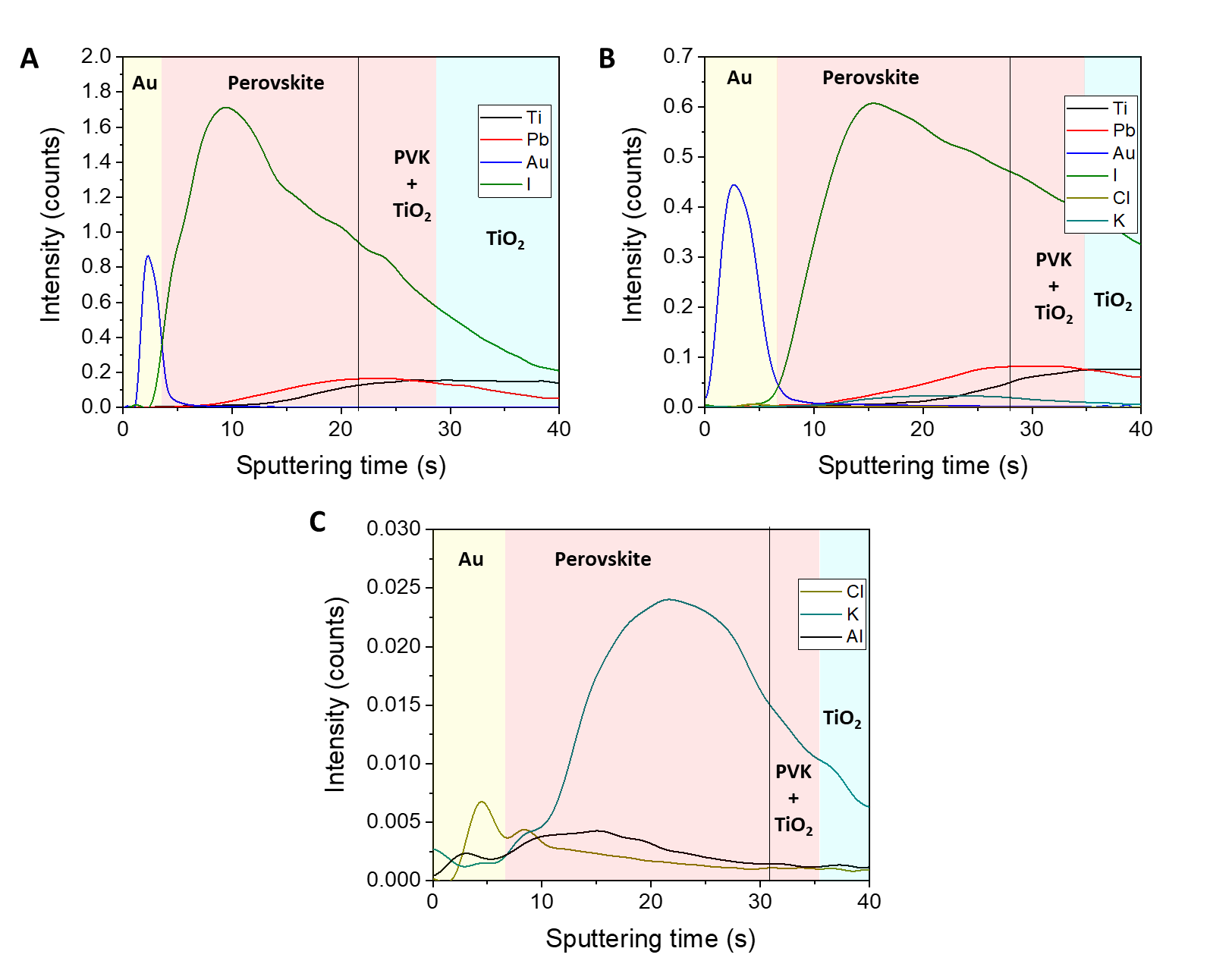


**Figure S4.** GD-OES profile of (A) pristine and (B) with KCl (5 mol%) d-MAPI-HEA devices. (C) GD-OES profile of Cl and K in d-MAPI-HEA KC5 doped final perovskite film. Al signal is displayed as a noise reference signal since Al is not present in the measured sample. The colored backgrounds identify the layers : yellow : gold back contact; Salmon : perovskite; blue : TiO_2_.


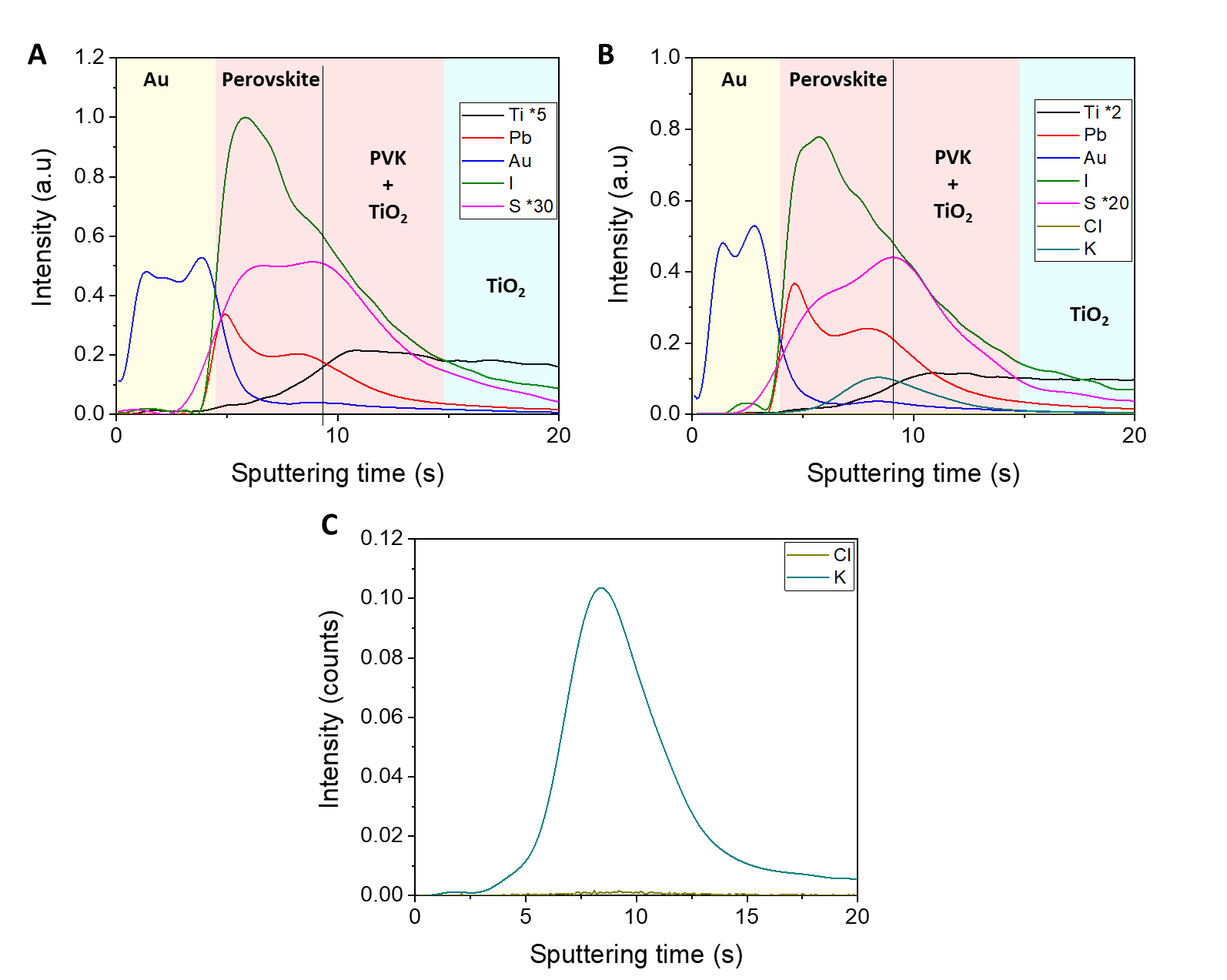


**Figure S5.** GD-OES profile of d-FAPI-TEA device (A) pristine and (B) with KCl (5 mol%). S signal correspond to TEA^+^. It was multiplied by 20 or 30 due to the low amount of TEAI added into the perovskite precursor solution resulting in a low amount of TEA^+^ cations present in the final perovskite film. (C) GD-OES profile of Cl and K in d-FAPI-TEA KC5 doped final perovskite film.


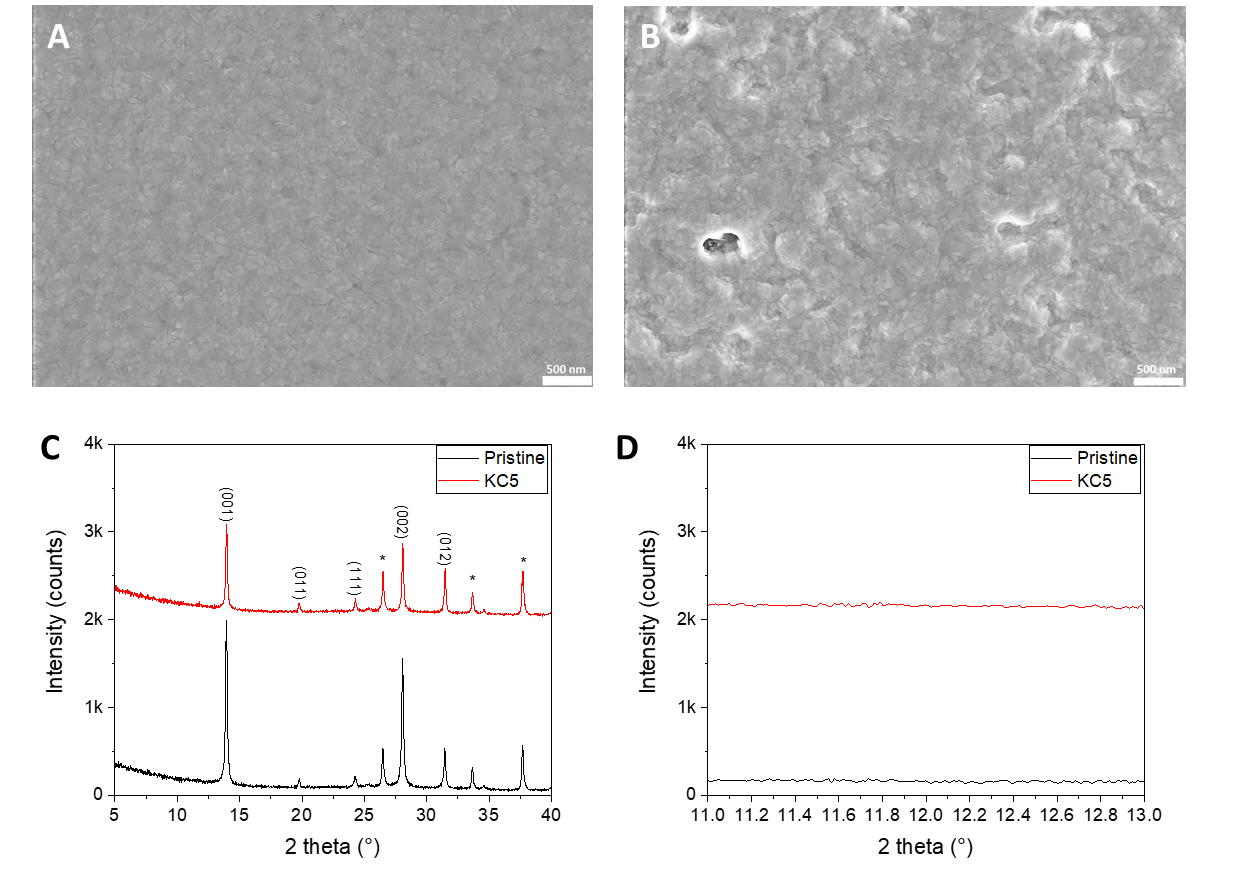


**Figure S6.** SEM top-view images of (A) pristine and (B) with KCl d-FAPI-TEA films. Scale bar: 500 nm. (C) XRD patterns of d-FAPI-TEA films with and without KCl. FTO peaks are indicated by *. (D) Same as (C) zoomed between 11° and 13°.

**
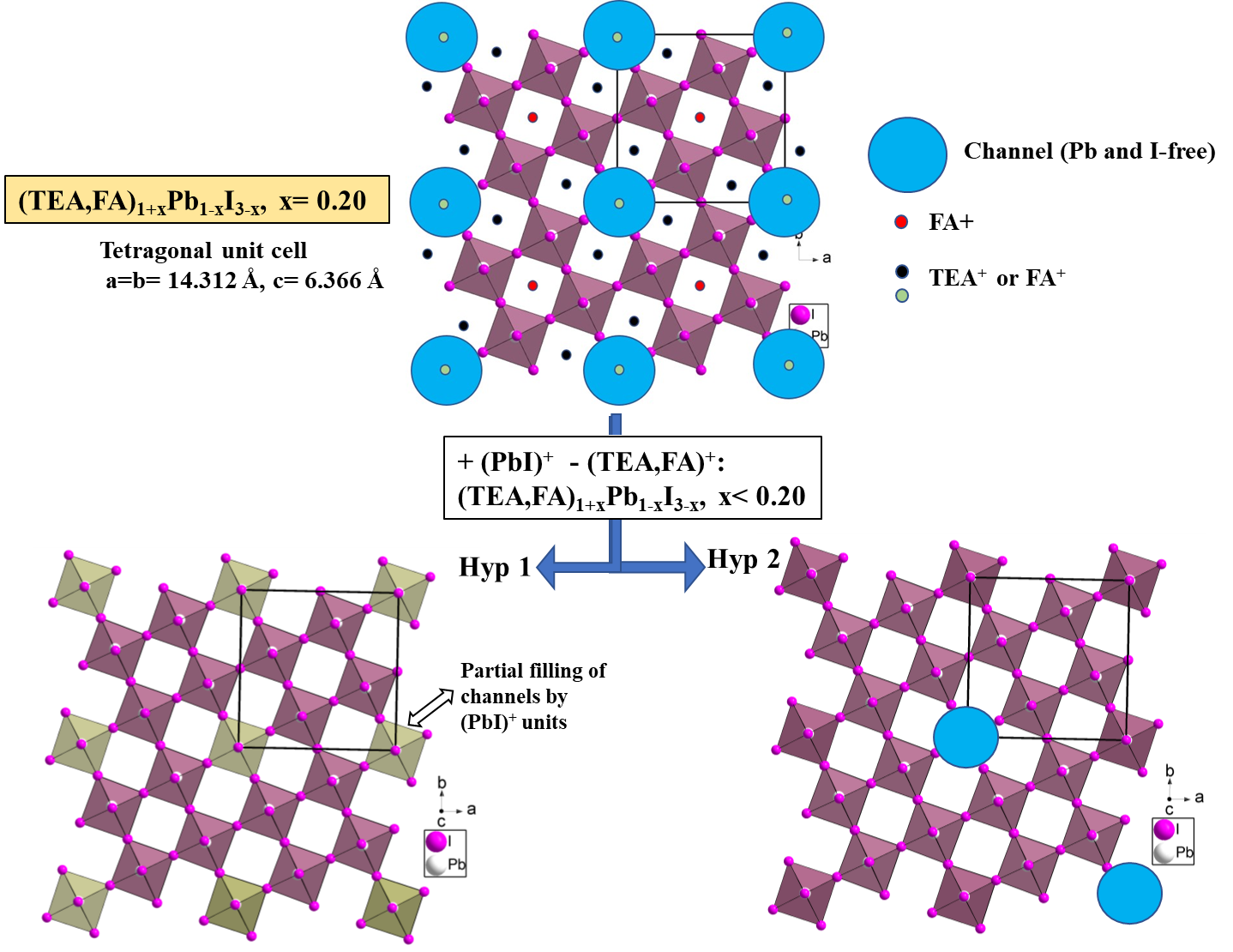
**

**Figure S7.** Structure of (TEA_,_FA)_1+x_Pb_1-x_I_3-x_ (x= 0.20) showing Pb and I-free channels (up), and model structures (Hyp1 and Hyp2) for compositions with x<0.20 : compared to the reference x=0.20 structure, all channels are partially filled by (PbI) units (Hyp 1), or some channels are fully filled by (PbI) units while some other remain Pb and I-free (Hyp 2).

**Table S5.** Exponential function used for Time-Resolved Photoluminescence (TRPL) curves fitting and extracted parameters.

| $\mathbf{y=}\boldsymbol{y}_{\boldsymbol{0}}\boldsymbol{+}\boldsymbol{A}_{\boldsymbol{1}}\exp\left( \boldsymbol{-}\frac{\boldsymbol{t}}{\boldsymbol{\tau}_{\boldsymbol{1}}} \right)\boldsymbol{+}\boldsymbol{A}_{\boldsymbol{2}}\mathbf{exp}\left( \boldsymbol{-}\frac{\boldsymbol{t}}{\boldsymbol{\tau}_{\boldsymbol{2}}} \right)\boldsymbol{+}\boldsymbol{A}_{\boldsymbol{3}}\mathbf{exp}\boldsymbol{(-}\frac{\boldsymbol{t}}{\boldsymbol{\tau}_{\boldsymbol{3}}}\boldsymbol{)}$ | | | | | | | |
| --- | --- | --- | --- | --- | --- | --- | --- |
| Sample | *y_0_* | *A_1_* | *τ_fast_* (ns) | *A_2_* | *τ_int_* (ns) | *A_3_* | *τ_slow_* (ns) |
| MAPbI_3_ | 4.14 10^-5^ | 0.19 | **0.72** | 0.58 | **5.00** | 0.23 | **15.62** |
| d-MAPI-HEA (KCl/NH_4_Cl) | 4.82 10^-4^ | 0.36 | **2.60** | 0.43 | **32.86** | 0.21 | **148.43** |
| FAPbI_3_ | 2.39 10^-4^ | 0.37 | **2.31** | 0.46 | **22.10** | 0.17 | **91.63** |
| d-FAPI-TEA | 1.42 10^-4^ | 0.28 | **12.29** | 0.70 | **3.21** | 0.02 | **65.06** |
